# Identifying links between athletic identity and risk factors related to youth sport participation

**DOI:** 10.3389/fpsyg.2024.1362614

**Published:** 2024-05-01

**Authors:** M. Minnat Choudhury, Ashley L. Erdman, Emily Stapleton, Emily Gale, Sophia Ulman

**Affiliations:** ^1^Movement Science Laboratory, Scottish Rite for Children, Frisco, TX, United States; ^2^Department of Psychology, Scottish Rite for Children, Frisco, TX, United States; ^3^Department of Psychiatry, University of Texas Southwestern, Dallas, TX, United States; ^4^Department of Orthopaedic Surgery, University of Texas Southwestern, Dallas, TX, United States

**Keywords:** athletic identity, adolescent athletes, sport specialization, youth sports, competition level, identity formation

## Abstract

**Introduction:**

The development of identity formation occurs during adolescence through experiences, ideals and principle. With greater accessibility to sports, recent trends have shown increased rates of sports specialization over the past decade in youth athletes. Athletic identity measures the strength an individual is tied to the athlete role and can be formed in conjunction to adolescent identity formation. More specialized youth athletes may have stronger ties to their athletic identity during their adolescent identity formation period.

**Methods:**

Youth basketball athletes were surveyed on specialization levels and athletic identity via the Athletic Identity Measurement Scale (AIMS), including three submeasures: social identity, exclusivity, and negative affectivity.

**Results:**

Participants showed stronger identification to social identity items and the weakest identification with exclusivity items. Athletes reporting more time spent playing their primary sport presented higher scores across all measures of athletic identity, and total athletic identity was stronger in athletes reporting specialization at an earlier age. Exclusivity and negative affectivity tended to increase with specialization level which may primarily be driven by specialized athletes choosing to quit non-primary sports.

**Discussion:**

Athletic identity may be worth noting as a psychological indicator of potential risk of injury. The long-term goal of this work is to provide the research and clinical community a greater understanding of a potential psychosocial risk factor as youth athletes continue specializing and spending more time training in a singular sport.

## 1 Introduction

In many instances, engagement with sports is the longest relationship children cultivate throughout their adolescence with 73% of adults indicating sports participation during childhood (Myer et al., 2015). While involvement in sports has been shown to provide an opportunity for children to learn behavioral, emotional, and physical skills (Robert Wood Johnson Foundation, 2015), several psychosocial aspects of sport participation remain unknown. Sports participation continues to increase among adolescents with an estimated 30 to 45 million youths between the ages of 6 and 18 participating in some form of athletics (Seefeldt et al., 1992). With greater accessibility to sports (Brenner, 2007), recent trends have also shown an increase in the training loads of youth athletes and sport specialization (or single-sport participation) over the past decade (Brenner, 2007; Jayanthi et al., 2013). Youth sports culture has evolved over time and the multi-faceted relationship adolescents form with sports is also adapting. Although recent literature has highlighted higher training volumes and single-sport specialization may be indicators of increased injury risk (Carder et al., 2020), psychosocial measures associated with sport participation characteristics, which may influence injury risk, have not yet been studied extensively.

Children begin forming their identity at an early age with adolescence as a pivotal period of identity formation based on lived experiences, beliefs and goals (Erikson, 1959). As they mature, the unique facets of one’s identity can influence the different roles they may fulfill throughout their life (Rattansi and Phoenix, 2005). These identities can stem from race/ethnicity, social class, and gender (Rattansi and Phoenix, 2005). These identities can also stem from activities adolescents dedicate their time to, which includes arts, studies and sports. Brewer et al. first coined the term athletic identity (AI) defined as a component of an individual’s concept of self and the degree the individual identifies with the athlete role (Brewer et al., 1993). Brewer et al. claims that individuals with strong ties to their athletic sense of self are more likely to have increased participation in sport, and this athletic identity may be associated with specific expectations or behaviors of the individual or the people within their environment (Brewer et al., 1993). For example, while increased athletic identity has not been proven to be an indicator of greater injury risk, athletes exhibiting a stronger relationship with their sport may exhibit a tendency to overtrain given their elevated commitment to the athlete role, which may result in an increased risk of sustaining an overuse injury (Myer et al., 2015). Athletic identity can be broken down into three subfactors: social identity, exclusivity, and negative affectivity. Social identity is the athletic identity component focused on the personal connection an individual has to the athlete role. Exclusivity is the component focused on the exclusiveness an individual’s identity is to the athlete role. Lastly, negative affectivity is the component focused on the emotional impact an individual has should an unwanted or negative sporting outcome occur. Athletic identity and its subfactors can help provide indications of the mindset the athlete operates under. Thus, a better understanding of the identity athletes may have established with their sport is crucial for healthcare providers as they can create more personalized injury prevention and/or rehabilitation strategies.

In conjunction with possible increased training loads, athletic identity may also play a role in an athlete’s decision to specialize in one sport. Recent trends show that sport specialization is becoming increasingly common in early to middle adolescence with some children specializing as early as 6 years old (Jayanthi et al., 2013). Specifically, recent literature has shown an overall increase in sports specialization with earlier onset, especially with the creation and development of select and travel leagues fostering year-round single-sport participation (Brenner, 2016). Additionally, specialization prior to puberty has been shown to be associated with higher injury rates and increased psychological stress (Jayanthi et al., 2013). As sport specialization has been linked to overtraining habits and a decline in sufficient rest via an offseason (Brenner, 2016), the increasing trend to specialize has generated debate about whether this intense dedication is deleterious or not for young athletes (Jayanthi et al., 2013). While some degree of specialization may be needed to develop an elite-level athletic skill set, there is no concrete evidence that it is necessary to achieve elite status (Jayanthi et al., 2013). Furthermore, as specialization has been established as a potential risk factor for injury, specialization may also give rise to higher degrees of athletic identity, subsequently increasing their risk for injury including overuse injuries.

Although previous studies have explored the connection between sports participation and injury risk in young athletes (Malisoux et al., 2013; Myer et al., 2015; Brenner, 2016; Post et al., 2017; Prieto-González et al., 2021), the interconnections between specialization, time and intensity factors, and athletic identity have not yet been analyzed. To our knowledge, no prior studies have explored the potential impact athletic identity may have on youth athletes as they develop their athlete skill set. A better understanding of this impact may allow sports medicine providers to better address injury risk concerns. Given strong athletic identities may promote increased participation in sports, unforeseen events such as an injury can disrupt the athletic identity component of an individual’s sense of self (Sparkes, 1998), and this disruption could be seriously detrimental to the recovery of an athlete. Therefore, the purpose of this study was to investigate whether a potential link exists between athletic identity and risk factors related to sport participation, including specialization status, competition level, and training volume, in healthy, youth athletes. We hypothesize that athletes with a greater degree of athletic identity will exhibit higher levels of sport specialization. A causal effect between athletic identity and increased risk of injury will not be identifiable but highlighting this potential relationship will provide greater clarity on the role athletic identity plays on injury risk in youth athletes.

## 2 Materials and methods

### 2.1 Participants

A convenience sample of youth basketball athletes between the ages of 9 and 18 years were asked to participate in a one-time testing session that involved completing surveys, which took place between October and December of 2022, in an effort to better understand the individual relationship between an athlete and their primary sport. Testing sessions were conducted at large youth basketball camps and skills training events hosted by the Dallas Mavs Academy. These events are open to any youth basketball player and thus athletes from all over the southern United States attend, from both rural and urban areas. Participants were excluded if they experienced recent musculoskeletal injury within the previous 3 months or were diagnosed with an orthopedic condition that would limit their ability to perform the required tasks. Participation in the study was voluntary with all participants providing informed assent and/or consent prior to participation. The study was specifically approved by a regional institutional review board.

### 2.2 Procedures

Participants completed an electronic survey on a tablet device while in-person at the event. All datapoints included for analysis were captured at this time. Demographic information such as age and sex were collected. Adolescent stages were determined based on age of testing. Early stage of adolescence was defined as 14 years old and younger, while the middle to late stage was defined in the range between 14 and 18 years old. A custom sports activity participation survey was developed, as seen in Appendix A, and provided to participants as well. This survey included subject-reported outcomes including the amount of time the athlete dedicates to their sport, highest achieved competition level for their primary sport, and whether they identified as a multi- or single-sport athlete. In addition, the sports activity participation survey included sport specialization question, as previously outlined by Jayanthi et al. (2013) which were used to determine the athlete’s exclusiveness and specialization in their self-identified primary sport. Lastly, the Athletic Identity and Measurement Scale (AIMS) questionnaire was also collected.

#### 2.2.1 Instruments

Specifically, three questions from the sports activity survey indicated specialization status. Participants chose either *yes* or *no* to each question. A value of 0 was assigned for each “*no*” response and a value of 1 was assigned for each “*yes*” response. The three values were summed together to determine the numerical score. A score of 0 or 1 indicated low specialization, 2 indicated moderate specialization, and 3 indicated high specialization.

The 10 statement Athletic Identity and Measurement Scale (AIMS) questionnaire was administered to measure the degree of strength in which the participant identified with the athlete role or the degree in which one devotes special attention to sports relative to other engagements or activities in life (Brewer et al., 1993). The questionnaire uses a seven-point Likert scale ranging from 1 (strongly disagree) to 7 (strongly agree). The total AIMS score was computed as the sum of values from all 10 statements in the survey. This overall measure was further broken down into three subsidiary factors of social identity, exclusivity, and negative affectivity. The social identity subfactor relates to the social identity theory where an individual’s self-concept is derived from a perceived membership in a relevant social group (Brewer et al., 1993). In this study, the social identity subfactor identifies the strength at which an individual’s self-worth is tied to their athletic identity. The social identity sub-score was the sum of scores from statements 1 through 3 and ranged from 3 to 21. The exclusivity subfactor identifies the strength at which an individual is tied to the athlete role relative to other identities and roles (Brewer et al., 1993). In essence, the exclusivity subfactor is the cognitive identity component for how exclusive the individual’s overall identity is tied to identifying as an athlete over other identities. The exclusivity sub-score is the sum of scores from statements 4 through 6 and 9 with scores ranging from 4 to 28. Lastly, the negative affectivity subfactor identifies the measure of the individual’s emotional state affecting their athlete role from unwanted sporting outcomes (Brewer et al., 1993). This factor measures the emotional identity connection with being an athlete. The negative affectivity sub-score is the sum of scores from statements 8 and 10 with scores ranging from 2 to 14.

### 2.3 Statistical analysis

Descriptive statistics including frequencies, means and standard deviations were calculated across all variables. Given significant Shapiro–Wilk tests of normality, non-parametric analyses were conducted. Specifically, Spearman correlations were performed to identify associations between AIMS measures and continuous variables including age, age at specialization, years playing basketball, months per year playing basketball and practices per week playing basketball. Differences in total AIMS score and AIMS sub-scores by binary variables were determined using Mann–Whitney U tests. This included sex, adolescent stage (early versus middle/late), competition level (recreation/school versus club/select), and whether the athlete reported quitting their non-primary sports, taking an offseason, playing more than one sport, and competing on a club and/or select team. Additionally, differences in AIMS scores by specialization level (low, moderate, high) were determined using Kruskal-Wallis tests. Subsequently, *post hoc* pairwise comparisons were performed, and Bonferroni corrections were applied for *post hoc* paired comparisons among the three levels of specialization (i.e., *α* = 0.017). Otherwise, statistical significance was concluded when *p* < 0.05.

## 3 Results

A total of 252 basketball athletes (99 females; age 13.8 ± 1.5 years, range 8.7–18.1 years) were included for analysis. The majority of participants reported basketball as their primary or main sport (227/252, 90.1%) and that they played basketball more than 8 months of the year (184/252, 73.0%). Additional sport participation measures, such as means or frequencies, are included in Table 1. Across all participants, the mean total AIMS score was 54.2 ± 11.2 out of 70 total points. As seen in Table 2, participants identified the least with questions 9 (‘Sports is the only important thing in my life’) and 6 (‘I need to participate in sport to feel good about myself’). In contrast, the most agreement was found with questions 1 (‘I consider myself an athlete’), 2 (‘I have many goals related to sport’), and 3 (‘Most of my friends are athletes’).

**Table 1:** Participant demographics and sport characteristics.

Variable	Value
	Mean ± SD
Age (years)	13.8 ± 1.5
Years in basketball	6.2 ± 3.1
Months active per year	9.3 ± 3.2
Practices per week	4.2 ± 2.0
Age at specialization (years)	9.4 ± 2.4
	N (%)
Sex (% female)	99 (39.3%)
Adolescent stage (% early)	151 (59.9%)
Quit non-primary sport(s) (% yes)	114 (45.2%)
Takes an offseason (% yes)	98 (38.9%)
Multisport participation (% yes)	140 (55.6%)
Competition level (% club/select)	4 (53.2%)
Specialization level	
*Low*	73 (29.0%)
*Moderate*	74 (29.4%)
*High*	105 (41.6%)

**Table 2:** AIMS individual question ratings (mean ± SD) for all participants.

AIMS question	AIMS score
1. I consider myself an athlete.	6.5 ± 1.3
2. I have many goals related to sport.	6.3 ± 1.3
3. Most of my friends are athletes.	6.0 ± 1.4
4. Sport is the most important part of my life.	5.5 ± 1.6
5. I spend more time thinking about sport than anything else.	5.2 ± 1.6
6. I need to participate in sport to feel good about myself.	4.7 ± 2.1
7. Other people see me mainly as an athlete.	5.9 ± 1.5
8. I feel bad about myself when I do poorly in sport.	5.4 ± 1.7
9. Sport is the only important thing in my life.	3.7 ± 2.0
10. I would be very depressed if I were injured and could not compete in sport.	5.0 ± 2.0

No significant correlations were found between AIMS measures and age. However, age at specialization was determined to be weakly correlated with total AIMS score (*r* = −0.212, *p* = 0.037). Specifically, the total AIMS score was higher in participants who reported specializing at an earlier age. For the remaining three continuous variables (total years, months per year, and practices per week playing basketball), weak to moderate correlations were also found with total AIMS score (*r* = 0.196–0.284, *p* ≤ 0.002), social identity (*r* = 0.274–0.350, *p* < 0.001), and exclusivity (*r* = 0.238–0.309, *p* < 0.001). Similarly, although not associated with years playing basketball, negative affectivity was significantly correlated to months per year (*r* = 0.237, *p* < 0.001) and practices per week (*r* = 0.231, *p* < 0.001) playing basketball.

No significant group differences were found in total AIMS or AIMS sub-scores by sex or adolescent stage (Table 3). Total AIMS score was significantly higher in participants who quit non-primary sports to focus on their primary sport (mean difference: 1.9, *p* = 0.031), did not report taking an offseason (mean difference: 5.3, *p* < 0.001), and participate on a club/select team (mean difference: 4.3, *p* = 0.016). Similar trends were observed for the AIMS sub-scores. Specifically, social identity was higher in participants who did not take an offseason (mean difference: 1.3, *p* = 0.006) and play on a club/select team (mean difference: 1.5, *p* = 0.019). Increased exclusivity was seen in participants who quit non-primary sports (mean difference: 1.6, *p =* 0.024) and did not take an offseason (mean difference: 2.5, *p* = 0.001). Lastly, negative affectivity was greater in participants who quit non-primary sports (mean difference: 1.1, *p* = 0.029), did not report taking an offseason (mean difference: 0.9, *p* = 0.004), and participate on a club/select team (mean difference: 1.0, *p* = 0.013).

**Table 3:** Means ± SDs of total AIMS and AIMS sub-scores.

Variable (N)	Total AIMS (Out of 70)	Social identity (Out of 21)	Exclusivity (Out of 28)	Negative affectivity (Out of 14)
ALL	54.2 ± 11.2	18.8 ± 3.6	19.1 ± 5.4	10.4 ± 3.2
*Sex*				
Female (99)	54.5 ± 10.7	18.6 ± 3.8	19.3 ± 4.8	10.7 ± 3.0
Male (153)	54.1 ± 11.5	18.9 ± 3.6	19.0 ± 5.8	10.2 ± 3.3
*Adolescent stage*				
Early (151)	54.4 ± 9.8	19.0 ± 3.2	19.1 ± 4.8	10.4 ± 3.2
Mid-Late (101)	54.0 ± 0.0	18.5 ± 4.2	19.1 ± 6.2	10.5 ± 3.1
*Quit non-primary sports*				
Yes (114)	**55.4 ± 12.1**	18.5 ± 4.3	**20.0 ± 5.5**	**11.1 ± 2.9**
No (1)	**53.5 ± 10.2**	19.2 ± 2.8	**18.4 ± 5.4**	**10.0 ± 3.3**
*Takes an offseason*				
Yes (98)	**51.5 ± 12.3**	**18.1 ± 4.4**	**17.8 ± 5.5**	**10.0 ± 3.0**
No (128)	**56.8 ± 9.7**	**19.4 ± 2.8**	**20.3 ± 5.3**	**10.9 ± 3.2**
*Multisport participation*				
Yes (140)	55.0 ± 10.5	19.0 ± 3.4	19.4 ± 5.2	10.5 ± 3.2
No (112)	53.2 ± 11.9	18.5 ± 3.9	18.7 ± 5.7	10.4 ± 3.1
*Competition level*				
Rec./School (118)	**51.9 ± 0.2**	**18.0 ± 4.5**	18.4 ± 6.1	**9.9 ± 3.4**
Club/Select (4)	**56.2 ± 8.5**	**19.5 ± 2.5**	19.7 ± 4.6	**10.9 ± 2.9**

Significant group differences noted in bold.

Regarding specialization level, age, and age at time of specialization did not significantly differ by specialization level (Table 4). However, the high specialization group did report playing basketball significantly longer than the low specialization group (mean difference: 1.4 years, *p* = 0.007). Additionally, both the moderate and high specialization groups reported playing basketball more months per year (mean difference: 3.2 and 3.6 months, respectively, *p* < 0.001) and practicing more times per week (mean difference: 0.9 and 1.3 times, respectively, *p* ≤ 0.004) compared to the low specialization group. For AIMS measures, total AIMS score (mean difference: 4.6, *p* = 0.001) and exclusivity (mean difference: 2.4, *p* = 0.008) significantly differed between the low and high specialization groups (Table 4). Though the moderate specialization group indicated AIMS scores that trended closely to that of the high specialization group, the AIMS score was not found to be significantly different between the low and moderate specialization groups. Alternatively, social identity significantly differed between the low and moderate specialization groups (mean difference: 2.0, *p* = 0.001). The high specialization group reported a social identity score between the low and moderate specialization group scores and did not significantly differ with either group. Finally, negative affectivity in the high specialization group significantly differed from both the low (mean difference: 1.3, *p* = 0.005) and moderate (mean difference: 1.3, *p* = 0.009) specialization groups.

**Table 4:** Means ± SDs of measures by specialization level.

Variable	Specialization level		
	Low (73)	Moderate (74)	High (105)
Age (years)	14.1 ± 1.5	0.7 ± 1.7	0.7 ± 1.4
Age at specialization	10.1 ± 2.7	8.7 ± 2.1	9.6 ± 2.4
Years in basketball	**5.3 ± 2.9**	6.3 ± 3.2	**6.7 ± 3.1** ^**L**^
Months/Year in Basketball	**6.9 ± 3.2**	**10.1 ± 2.8** ^**L**^	**10.5 ± 2.6** ^**L**^
Practices/Week in Basketball	**3.4 ± 1.8**	**4.3 ± 1.9** ^**L**^	**4.7 ± 2.0** ^**L**^
*AIMS Measures*			
Total AIMS	**51.1 ± 12.1**	55.2 ± 7.9	**55.7 ± 12.1** ^**L**^
Social Identity	**17.9 ± 4.1**	**19.9 ± 1.5** ^**L**^	18.6 ± 4.2
Exclusivity	**17.6 ± 5.6**	19.3 ± 4.7	**20.0 ± 5.6** ^**L**^
Negative Affectivity	**9.9 ± 3.2**	**9.9 ± 3.4**	**11.2 ± 2.9** ^**L,M**^

Significant group differences noted in bold. Paired differences between specialization groups indicated with superscripts. Specifically, differences between the Low specialization group are noted with superscript L, and differences between the Moderate specialization group are noted with superscript M.

## 4 Discussion

The purpose of the current study was to explore the potential link between athletic identity and risk factors related to sport participation, including specialization status, competition level, and training volume in a healthy, youth athlete population. Across the ten statements of the AIMS questionnaire, participants showed stronger identification with those relating to social identity and the weakest identification with the statements relating to exclusivity. Total athletic identity was stronger in athletes reporting specialization at an earlier age. Additionally, key differences between moderately and highly specialized athletes compared to low specialized athletes were seen in total athletic identity, exclusivity, and negative affectivity. Overall, athletic identity measures tended to increase with specialization level, except social identity which deviated from this trend.

Similar work by McGinley et al. focused on athletic identity in a youth patient population found that athletes between the ages of 10 and 24 who had recently experienced an anterior cruciate ligament (ACL) injury more strongly agreed with the social identity statements from the AIMS questionnaire (McGinley et al., 2022), comparable to the findings presented in the current study. Specifically, across both study populations, statements with the highest scoring agreement were ‘I consider myself an athlete’, ‘I have many goals related to sport’, and ‘Most of my friends are athletes’. The same cohort of ACL-injured athletes also identified the least with the statements on exclusivity of their athletic identity. Both study populations primarily disagreed with the statements ‘I need to participate in sport to feel good about myself’ and ‘Sport is the only important thing in my life’. Interestingly, although similar trends were found, total AIMS was found to be notably lower in the patient population (49.4 ± 11.9) compared to the healthy population (54.2 ± 11.2) included in the current study. This may be due to the age difference between study populations as the patient group was older than the healthy group presented in the current study (15.9 vs. 13.8 years). However, Houle et al. found that athletic identity increased with age, on average, as athletes reported higher AIMS scores at the age of 15 compared to when they were 10 years old (Houle et al., 2010). Therefore, while the responses across AIMS statements remain similar, it may be possible that experiencing an injury reduces an athlete’s overall athletic identity. Future investigation is warranted to determine how time loss from sport impacts a young athlete’s identity.

Findings from the current study suggest that while an athlete’s social connection to being an athlete is strong, their self-identity is not strongly singularly faceted with being an athlete. This theory is supported in the current study by the findings observed with competition level. Specifically, athletic identity in athletes playing recreationally, either in the community or on their school team, significantly differed compared to athletes who reported playing on a club/select team. Lower competition level was found to be associated with reduced overall athletic identity (total AIMS), social identity, and negative affectivity. Thus, while higher competition levels often require elevated training volume and dedication to sport, playing in a more competitive and challenging environment may result in a closer identification with the athlete role both socially and emotionally, but not necessarily influence the individual’s development of other facets of self-identity. While they still identify with the athlete role given their overall AIMS score, they may continue to have a diversification of self-identity given their lack of exclusivity to the athlete role. Pot et al. conducted a study across one school year focused specifically on organized soccer competition within elementary schools in the Netherlands, where students (10–12 years old) played once a week on their school’s team. Their primary aim was to determine whether the students’ sport identity via AIMS varied with participation in competition. Students in the study who participated in competition for the full school year displayed higher total AIMS compared to those who did not participate or dropped out prior to the year-mark. Additionally, most of the students who continued participation in competition also reported playing in a sports club. Thus, the authors suggested that sporting activities beyond organized school sports may also contribute to an athlete’s sense of athletic identity, which corroborates the results found in the current study (Pot et al., 2014).

Further, a second study conducted by Mitchel et al. explored how various playing levels affected athletic identity measures in male elite youth English footballers between the ages of 16 and 18 years old across multiple football clubs and multiple leagues (Mitchell et al., 2014). Across the playing levels, no significant differences were reported in total athletic identity, but this may be due to the homogeneity of the cohort tested as all athletes were teenage males playing at an elite level. Alternatively, exclusivity did significantly differ between playing levels suggesting that the football environment and culture in the individual football clubs and leagues may explain exclusivity to an athletic identity in players and influence the strength of the relationship between the athletes’ self-identity and their role as an athlete (Mitchell et al., 2014). These findings relate to the current study in that exclusivity did significantly differ between competition playing levels, which is potentially explained by the general environment and culture curated at the various competition levels of youth basketball. For example, athletes who compete at higher levels may be more likely to singularly identify with the athlete role and exhibit decreased diversification in their identity compared to other developmentally appropriate roles, such as being a student.

In addition to competition level, another factor which may influence athletic identity is specialization status. In the current study, on average, AIMS measures were elevated with higher levels of specialization on average. Given specialized athletes’ tendency to have high training volumes, as shown in the current study and by recent literature (Moesch et al., 2011; Myer et al., 2015; Brenner et al., 2019), a greater portion of their life experience is centered on sport, which can have an increased influence on their development (Brenner et al., 2019). Although only weak correlations were found between training volume and athletic identity, differences in training volume between specialization groups mirrored the trends observed in AIMS scores, specifically between high and moderate specialization groups and the low specialization group. However, training volume did not significantly differ between moderate and highly specialized athletes. Thus, while training volume may explain differences with the low specialization group, these measures may not be the only influential factors on athletic identity, especially in higher levels of specialization. In the current study, specialization status was determined based on whether the athletes reported having a primary sport, playing their primary sport more than 8 months out of the year (versus taking an offseason), and quitting secondary sports to focus on their primary sport. Thus, it may be possible that singular aspects of specialization more directly relate to athletic identity. For example, social identity only displayed a significant difference with whether the athlete took an offseason whereas overall athletic identity (total AIMS), exclusivity, and negative affectivity were higher in participants that took an offseason and quit non-primary sports. This may indicate single-sport specialization does not strongly influence an athlete’s social connection to their sport, but rather is primarily driven by the cumulative time spent in that sport. Alternatively, the degree to which an athlete’s self-identity and emotional state is intertwined with their primary sport is more likely impacted by the athlete’s decision to isolate their focus to one sport by quitting non-primary sports.

The current study also found that participants who reported specializing at an earlier age exhibited higher overall athletic identity via total AIMS score. Although a common misconception, Jayanthi et al. (2013) identified that early sports specialization and therefore early intense training is not necessarily essential to attaining an elite level at sports. Specifically, they found that across high-level basketball athletes, the greater number of activities the athlete experienced and practiced in their development years (0–12 years), the less sport-specific practices were needed to gain expertise in their sport (Baker et al., 2003). This may indicate that these athletes engaged in adequate independent exploration of identity throughout adolescence and committing to that identity and goals, such as becoming an elite athlete. With higher exclusivity and negative affectivity seen in athletes who chose to isolate and specialize in a single sport, young athletes with more diverse identities may bolster themselves from negative psychological and physical outcomes long-term, since specialization prior to puberty has been shown to be associated with higher injury rates and increased psychological stress (Baker et al., 2003).

Erik Erikson, a developmental psychologist known for his seminal theory of psychosocial development and identity, identified that adolescence is the period in the human life cycle an individual establishes and forms their sense of personal identity (Erikson, 1959). During this time period and especially during the teenage years, identity is especially malleable during adolescence given the external influences from peer groups and family and a desire externally from groups such as peers and family along with their expectations and internally with goal aspiration including perseverance, skill level and ability (Erikson, 1959). Athletes specializing at younger ages are highly influenced by family and people they admire, which can lead to engagement in a sport familiar to them or that parents approve or push. If the child decides to specialize during the pre-adolescence phase, they may isolate themselves though eliminating non-primary sport involvement. This can cause identity foreclosure in adolescence, where they have high commitment to their identity without exploring alternative identities (Marcia, 1966). A study performed by McQuown Linnemeyer and Brown (2010) found that athletes reported higher identity foreclosure than non-athlete peers. Research conducted by Petipas indicated that athletes are at risk for identity foreclosure due to the demands of sport participation and emphasis on conformity and compliance in sport environments, hindering the athlete’s ability and/or desire to explore alternative identities and interests (Petitpas and France, 2012).

This study does have a few limitations that should be noted. Given that this study is one of the first to report normative values of athletic identity in a healthy, youth athlete population (specifically via the AIMS questionnaire), it is difficult for the authors to determine whether a specific mean difference is meaningful. Future work is needed to compare AIMS responses to a more global assessment of athletic identity and conduct sensitivity analyses on the tool such that researchers or clinicians may interpret a meaningful change in scores. Additionally, the majority of cohort tested in this study were between 12 to 15 years old, at the cusp of early to middle adolescence. As the development of self-identity and the relationship between an athlete and their sport evolves dramatically throughout adolescence, the findings presented here may not be applicable for a younger athlete population. Further work should include youth in the early adolescent stage as well as consider changes in athletic identity throughout the stages of adolescence. Lastly, all athletes included in the current study reported basketball as their primary sport. As population trends or interpretation of specialization status, competition level, and training volume may differ across sports, it is challenging to interpolate the results found in the current study to other sports, especially as sport type may also influence athletic identity (e.g., team sports, season-dependent sports such as water or winter sports). Further investigation into the athletic identity should span across age, sex, time, and sport in order to capture a more comprehensive understanding of the psychosocial impacts of sport on youth athletes. Similarly, while competition level and training volume were captured, the intensity of training was not explicitly asked. As training intensity, alongside duration of training, may be a strong indicator for injury, future work should consider the type of training and level of intensity the athlete regularly experience.

In conclusion, athletes reported stronger identification with social identity and the weakest agreement with statements contributing to exclusivity. Across all measures of athletic identity, elevated scores were observed in athletes that reported spending more time playing basketball, their primary sport. Additionally, exclusivity and negative affectivity tended to increase with specialization level which may primarily be driven by specialized athletes choosing to quit non-primary sports. Future work should investigate athletic identity in healthy youth athletes playing a variety of sports, both team and single-athlete sports, and within a younger youth population. Through additional investigation across a more diverse population, meaningful differences in AIMS scores between groups and over time may be better interpreted. The long-term goal of this work is to provide the research and clinical community with a better understanding of a potential psychosocial risk factor as youth athletes continue to specialize and spend more time training in sport. Athletic identity may be worth noting as a psychological indicator of potential risk of injury.

## Data availability statement

The raw data supporting the conclusions of this article will be made available by the authors upon reasonable request without undue reservation.

## Ethics statement

The studies involving humans were approved by University of Texas Southwestern Institutional Review Board. The studies were conducted in accordance with the local legislation and institutional requirements. Written informed consent for participation in this study was provided by the participants' legal guardians/next of kin.

## Author contributions

MC: Conceptualization, Data curation, Formal analysis, Investigation, Methodology, Visualization, Writing – original draft, Writing – review & editing. AE: Data curation, Formal analysis, Investigation, Methodology, Writing – review & editing. ES: Conceptualization, Formal analysis, Investigation, Methodology, Writing – review & editing. EG: Conceptualization, Formal analysis, Investigation, Methodology, Writing – review & editing. SU: Conceptualization, Data curation, Formal analysis, Investigation, Methodology, Project administration, Resources, Supervision, Writing – review & editing.

## Appendix

Appendix A – Sports Activity Participation Survey

1. Is basketball your primary sport?
2. How many years have you played basketball?
3. At what level do you play basketball?
4. How many months per year do you play basketball?
5. How many times do you practice per week for at least an hour?
6. What is your primary position?
7. Do you feel like you have a main sport?
8. What is your main sport?
9. Do you spend more than 8 months out of the year participating in basketball?
10. Did you quit other sports to participate in basketball?
11. Do you take an offseason from basketball?
12. Do you play other sports competitively?
  - If yes, what age did you decide to only participate in basketball?
13. Have you ever had an injury to your knee?
14. Have you had any sports-related injuries in the last year?
